# In vivo human retinal swept source optical coherence tomography and angiography at 830 nm with a CMOS compatible photonic integrated circuit

**DOI:** 10.1038/s41598-021-00637-4

**Published:** 2021-10-26

**Authors:** Elisabet A. Rank, Stefan Nevlacsil, Paul Muellner, Rainer Hainberger, Matthias Salas, Stefan Gloor, Marcus Duelk, Martin Sagmeister, Jochen Kraft, Rainer A. Leitgeb, Wolfgang Drexler

**Affiliations:** 1 Center for Medical Physics and Biomedical Engineering, Medical University of Vienna, 1090 Vienna, Austria; 2 AIT Austrian Institute of Technology GmbH, 1210 Vienna, Austria; 3 EXALOS AG, 8952 Schlieren, Switzerland; 4 ams AG, 8141 Premstaetten, Austria

**Keywords:** Integrated optics, Silicon photonics, Three-dimensional imaging

## Abstract

Photonic integrated circuits (PIC) provide promising functionalities to significantly reduce the size and costs of optical coherence tomography (OCT) systems. This paper presents an imaging platform operating at a center wavelength of 830 nm for ophthalmic application using PIC-based swept source OCT. An on-chip Mach–Zehnder interferometer (MZI) configuration, which comprises an input power splitter, polarization beam splitters in the sample and the reference arm, and a 50/50 coupler for signal interference represents the core element of the system with a footprint of only $(12 \times 5)\;{\text {mm}}^2$. The system achieves 94 dB imaging sensitivity with 750 $\upmu$W on the sample, 50 kHz imaging speed and 5.5 $\upmu$m axial resolution (in soft tissue). With this setup, in vivo human retinal imaging of healthy subjects was performed producing B-scans, three-dimensional renderings as well as OCT angiography. These promising results are significant prerequisites for further integration of optical and electronic building blocks on a single swept source-OCT PIC.

## Introduction

Optical coherence tomography (OCT) has become the most successful imaging technique to non-invasively visualize the intraretinal layers^1^. Rapid improvement of imaging speed and quality (resolution/sampling) led to widespread clinical acceptance, and OCT is now considered to be the gold standard for diagnosing ophthalmic diseases^2^. However, due to a relatively large footprint of approximately a square meter and high investment costs as compared to other ophthalmic diagnostic devices (high end) OCT systems are typically limited to clinical settings such as hospital clinics and optometry practices^3^. OCT is a fast and easy-to-use diagnostic tool for early detection of retinal diseases that can also be of great benefit in general practices. With a reduction of size and cost, OCT devices may be used even more widely and thereby improve retinal care—in particular in low resource settings. Photonic integrated circuits (PIC) are a promising technology to achieve reduction of both size and costs of OCT systems in addition to enabling quasi-maintenance free operation. PICs have a typical footprint of less than 1 ${\text {cm}}^2$ and are therefore significantly smaller than fiber or free-space optical systems. Furthermore, PICs can be co-integrated with CMOS-based electronics as PIC fabrication uses the same machines and techniques as the CMOS industry. Multiple opto-electronic PICs can be produced on a single wafer in parallel, which will significantly reduce the costs of such devices.

A core element of a swept source OCT (SS-OCT) device is an interferometer, typically either in Michelson or in Mach–Zehnder configuration^4^. The output power of a swept source is divided into sample and reference arm power. Backreflected light from both arms is redirected towards the coupler with a splitting ratio of 50/50. The interfered light is then forwarded to a dual balanced detector, which acquires the signals using two photodiodes.

Various PIC-based interferometers for OCT application have been reported in literature.

Yurtsever et al. demonstrated the development of an on-chip Michelson interferometer at 1500 nm with a system sensitivity of 25 dB^5,6^. Later, they implemented a broadband adiabatic coupler for reduced wavelength dependence of the splitting ratio and achieved a system sensitivity of 62 dB with 3 mW on the sample^7^. Using software-based dispersion compensation they optimized the axial resolution of the system to 25 $\upmu$m and demonstrated a tomogram of a three-layered scotch tape (a hundred times averaged). Nguyen et al. reported a Mach-Zehnder interferometer (MZI) for 1300 nm with a system sensitivity of 80 dB and an axial resolution of 13 $\upmu$m^8^. Wang et al. combined an on-chip polarization splitter, a polarization rotator and a photodetector including integrated transimpedance amplifiers^9^. The sample and reference inputs were separated in X- and Y-polarization channels, which were then forwarded to four separate photodetectors. With this system they achieved a system sensitivity of 94 dB with 26 mW on the sample and demonstrated full-range OCT on the inner human lip in vivo (B-scan) as well as polarization diversity catheter based OCT on a swine artery (B-scan and enface) and polarization sensitive OCT of an ex-vivo human artery. Schneider et al. demonstrated an on-chip splitter with an on-chip delay line^10^. With an on-chip photodiode they achieved a sensitivity of over 40 dB and an axial resolution of 100 $\upmu$m at 1300 nm with which they imaged a piece of pumice (B-scan). Further integration was shown by Schneider et al. with two on-chip multimode interferometers: one with an external reference arm (OCText), and the other one with an on-chip reference arm (OCTint)^11^. Germanium photodiodes with a 20 GHz bandwidth were integrated as well. With these systems, sensitivities of 64 dB and 53 dB were achieved for OCText and OCTint, respectively, and B-scans of Scotch tape as well as enface images of pumice and a decayed leaf were shown. Van Leeuwen et al. reported an MZI with an on-chip reference arm for 1550 nm^12^. They achieved a system sensitivity of 83 dB and an axial resolution of 15.2 $\upmu$m. To the best of our knowledge, all reported on-chip interferometers for SS-OCT application are in the telecom wavelength region (1300–1550 nm), which are not ideal for human retinal imaging due to increased water absorption of ocular media in these wavelength ranges.

For retinal imaging, the thickness of the retina does usually not exceed 300–500 $\upmu$m and layer visualization on the order of  10–15 $\upmu$m is required^13^. High system sensitivity is desired, but low system sensitivities can be compensated for by image averaging, reduction of acquisition speed or more power on the sample. Involuntary movement of the eyeball is a challenge for image averaging, requiring motion correction algorithms to be implemented. Furthermore, due to the movement of the eye ball, imaging speed has to be fast enough for both volume acquisition and OCT angiography (OCTA) calculation. Finally, laser safety standards have to be followed to guarantee the patient’s health, which limits the allowed power on the eye. An OCT system for retinal imaging therefore needs to fulfill the following requirements: axial resolution below 10 $\upmu$m, imaging speeds higher than 20 kHz for volume and OCTA acquisition, low power on the sample (750 $\upmu$W in retinal imaging at 800 nm^14^), and good sensitivity (above 90 dB). In a recent study we showed, to the best of our knowledge, the first in vivo human retinal tomograms using two designs of PIC based arrayed waveguide gratings (AWG) for spectral domain OCT (SD-OCT), in which the above-mentioned parameters were of critical importance as well^15^. While an imaging speed of 67 kHz enabled the possibility to extract angiographic OCT data from the volumes, the sensitivity of the system dropped to below 90 dB and hence OCTA was challenging. By decreasing the imaging speed by a factor of two, the tomogram contrast improved but correction for eyeball movement within a volume became more challenging. Although all major retinal layers could be distinguished with an axial resolution of 10.7 $\upmu$m with the small bandwidth design (AWG design 1) a finer resolution was desirable. Tomograms with a resolution of 6.5 $\upmu$m were produced with AWG design 2, which had a broader bandwidth.

Nevlacsil et al. recently proposed a concept for an on-chip multi-channel SS-OCT system, in which multiple sample beams are used to increase the effective acquisition speed while maintaining the system sensitivity of a standard single beam system^16^. Unlike in fiber or free-space optics, PICs have the inherent advantage that multiple functions can be integrated on a single chip without the need for additional components or additional efforts for alignment or maintenance. Furthermore, SS-OCT has the advantage that the detection is realized with a single pair of dual balanced photodiodes and the implementation of multiple sample and detection arms is therefore less complex, compared to a multichannel on-chip SD-OCT system, where multiple AWGs would be needed.

In order to take advantage of the possibilities offered by PIC technology, the individual components need first to be developed and evaluated separately before they can be combined to a more complex configuration on a single chip. In this paper, an on-chip MZI as core of a swept source OCT system for in vivo ophthalmic application centered at 830 nm is presented.

**Figure 1:**
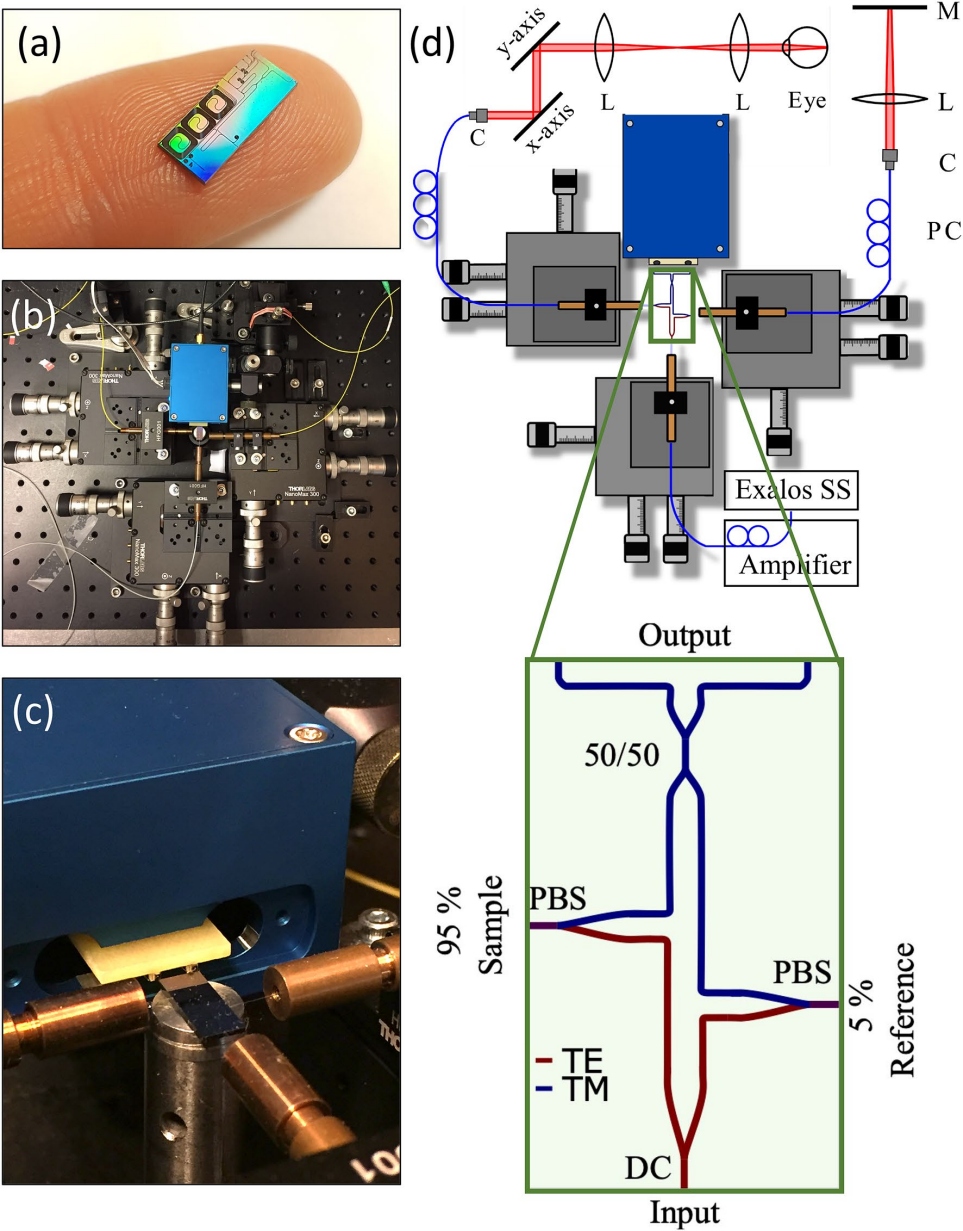
Measurement setup using an interferometer on a PIC: (**a**) Photograph of the PIC with the on-chip MZI. Additional test structures are visible, which were not part for the OCT setup. (**b**) Photograph of the PIC-based OCT setup, excluding the sample and reference arm and (**c**) close up photograph of the setup with the PIC surrounded by three fibers and a dual balanced receiver, where the photodiodes are mounted to the yellow extension plate. (**d**) Schematics of the setup design and the PIC layout: light from a swept source is amplified using a booster amplifier. A fiber with a straight cleaved fiber tip is aligned to the input port of the PIC, which guides the light to a on-chip power splitter (DC, directional coupler). 95% of the light are forwarded to the sample arm and 5% are forwarded to the reference arm. The light exiting the PIC is collected with straight cleaved fibers and connected to free-space sample and reference arm. Back-reflected light is coupled back onto the chip and forwarded to an on-chip 50:50 coupler via the *PBS* polarization beam splitter. The interfered light exits the PIC on the end-facet and is directly collected with a dual balanced receiver. *PC* polarization controller, *C* fiber collimator, *L* lens, *M* mirror, *TE*, *TM* TE-like and TM-like mode.

## Results

Figure 1 gives an overview of the developed setup incorporating the on-chip MZI. Light is coupled to and from the PIC using single mode fibres on the MZI input, sample and reference arm. Back-reflected light from the free-space sample and reference arms is interferred in the on-chip 50/50 coupler, exits the PIC at the end facet and is acquired in free-space using a customized dual balanced receiver. For more details on the system see the “Methods” section.

### System characterization

The efficiency of coupling light from fiber to PIC and vice versa determines the required laser power and also influences the system performance in terms of sensitivity in this PIC-based setup. Table 1 summarizes the measured insertion losses (IL), which include coupling, propagation and photonic building block losses. The booster amplifier was set to emit 11.9 mW ex fiber and the fiber was aligned to the input waveguide of the PIC. After aligning the sample and reference arm fiber the maximum powers that could be achieved were 2.5 mW and 0.1 mW, respectively. The power ratio of the two arms translates into the power splitter ratio of approximately 95/5. After accounting for the coupling ratio of the input power splitter IL values of 6.7 dB were determined. On the path towards the detector about half the insertion loss was measured resulting from the power being measured directly with a photodetector rather than coupling the light back into a fiber. From other measurements it was determined that the losses are mainly dominated by the coupling losses, which have an upper limit of about 3.4 dB in this case. The sample arm fiber was connected to the booster amplifier, providing 11.9 mW to the sample arm port. The powers exiting the PIC at the two dual balanced waveguides were measured in free space to be 2.6 mW and 2.8 mW, which calculates to a splitting ratio of approximately 48/52 and IL of 3.4 dB.

**Table 1:** Measured insertion losses (IL) of the fiber to PIC coupling in this setup: the measured input power as well as the measured output power are shown.

Coupling position	IN (mW)	OUT (mW)	C (dB)	SR (%)	IL (dB)
Input (ex fiber)–Samp. (ex fiber)	11.90	2.45	− 0.21	95.33	6.66
Input (ex fiber)–Ref. (ex fiber)	11.90	0.12	− 13.31	4.67	6.66
Samp. (ex fiber)–DB channel 1	11.90	2.62	− 3.22	47.64	3.35
Samp. (ex fiber)–DB channel 2	11.90	2.88	− 2.81	52.36	3.35

Splitting ratio in dB (*C*) and in $\%$ (*SR*) are calculated from measured values. In- and output powers (*IN*, *OUT*) ex fiber or in free space, resulting insertion losses *IL* representing the coupling, propagation and building block losses for two (input-sample and input-reference) and one (sample-dual balanced (*DB)* ports) fiber-PIC events, respectively.

**Figure 2:**
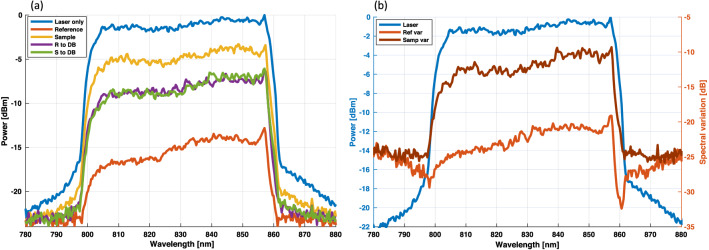
Laser spectra measured ex fiber compared to the PIC output ports . (**a**) Individual spectra measured ex-fiber and for different PIC input/output port combinations i.e. Sample = PIC input to sample port, Reference = PIC input to reference port, S/R to DB = sample/reference port to one balanced detection port. (**b**) Spectral variation caused by the propagation through the PIC in comparison to the laser spectrum.

Figure 2 shows the spectral transmittance through the PIC in comparison to the laser spectrum ex fiber. As in the power loss measurements above, the fiber was aligned to the input port and the spectra were acquired at the sample and reference arm, respectively. In this measurement the spectrum was acquired using a spectrum analyzer (USB2000+, OceanOptics, USA) in free-space rather than coupling back into a fiber. The laser fiber was then aligned to the sample and reference-ports, respectively, and the spectra of one output port after the 50/50 coupler was acquired. Figure 2a summarizes the individual spectra measured ex fiber and at the different PIC output ports. The spectral variation of the initial laser spectrum caused by the propagation through the PIC is shown in Fig. 2b. It was calculated by multiplying the spectrum measured at the sample (reference) port, with the laser fiber aligned to the PIC input port, with the spectrum at the balanced detection port, with the laser fiber aligned to the sample (reference) port, and dividing the result by the laser spectrum ex fiber.

**Figure 3:**
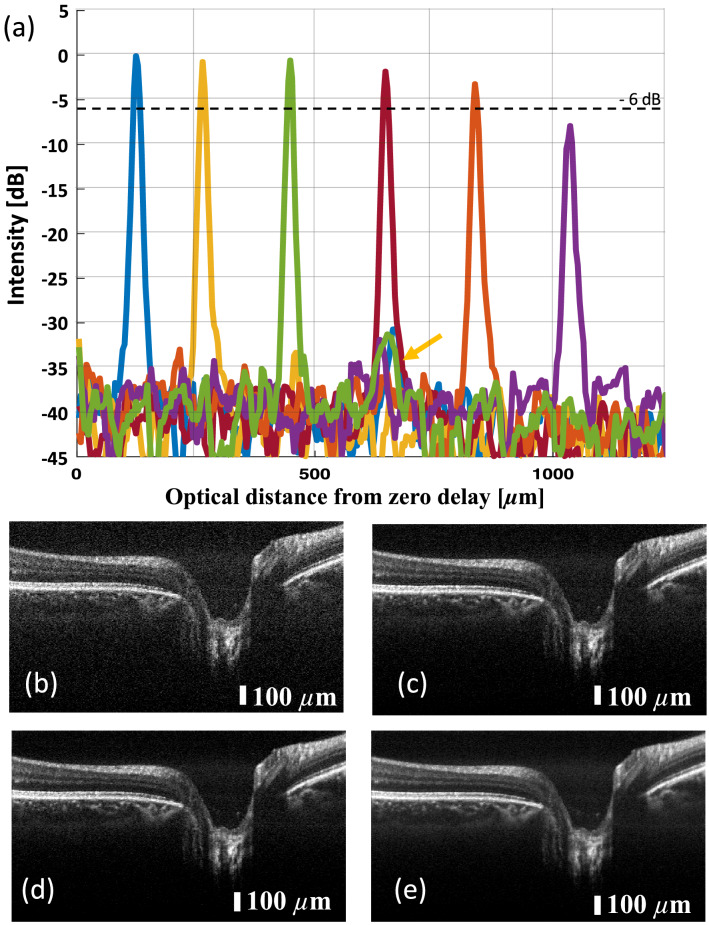
Characterization measurements of the on-chip MZI OCT setup: (**a**) shows the signal roll-off in depth of the system. An electrical noise signal can be seen at a depth of $\tilde{600}~\upmu {\text {m}}$ (yellow arrow), which is not constant over time, hence it does not appear in all A-scans. (**b**) shows a non-averaged tomogram of a retina in vivo. (**c**–**e**) show the same data set averaged three, five and ten times, respectively.

The system sensitivity was measured by adjusting the amplification to achieve 750 $\upmu$W on the sample, which was the power used for in vivo imaging as well. The reflectivity of a mirror as a sample was attenuated by a neutral density filter and the SNR of the point spread function was measured. By introducing the neutral density filter with a measured double pass attenuation of 52 dB the sensitivity was determined: An SNR of 42.2 dB was measured, which adds up to 94.2 dB system sensitivity. From the point spread function the axial resolution was calculated to be 7.5 $\upmu$m, which corresponds to 5.5 $\upmu$m in soft tissue, assuming a refractive index of 1.3549^17^. The theoretical axial resolution was calculated to be be 5.4 $\upmu$m (830 nm central wavelength, 56 nm 3-dB bandwidth). Figure 3a shows the signal roll-off in depth of the system. A 6 dB roll-off was measured at approximately 1 mm.

### In-vivo retinal imaging

Healthy subjects aged between 30 and 35 were imaged using the PIC-based OCT system. The whole study was approved by the institutional ethics committee of the Medical University of Vienna and following the tenets of the Declaration of Helsinki. Informed consent of the volunteers was obtained after explaining the form and nature of the measurements. Data was acquired in undilated eyes comprising 800 A-scans per B-scan. For volume acquisition, 400 A-scans per B-scan and 400 B-scans with a repetition of four (for OCTA) were acquired. Figure 3b shows a non-averaged tomogram. Signal averaging is a commonly used method to enhance the dynamic range in a tomogram. Figure 3c–e show the same B-scan location with increasing number of averaged B-scans, i.e. three times in Fig. 3c, five times in Fig. 3d and ten times in Fig. 3e.

**Figure 4:**
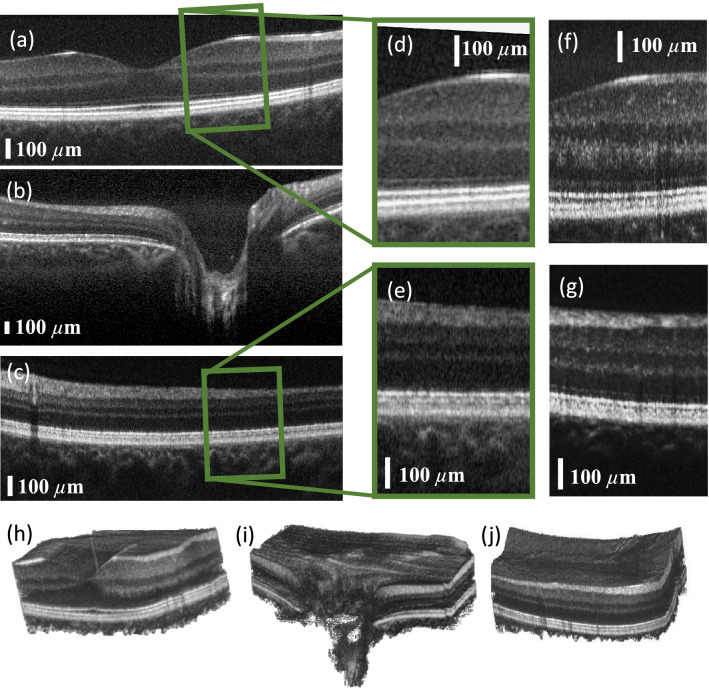
In vivo tomograms of a healthy retina: (**a**) area of the foveal pit, where the external limiting membrane indicates good axial resolution as well as good dynamic range in the tomogram; (**b**) the optical nerve head depression. Here, the choroid/sclera junction indicates good penetration depth. (**c**) Peripheral region of the retina with a vessel shadowing the layers below; (**f**,**g**) were acquired with configuration 2, which generates tomograms with higher lateral resolution and therefore contrasts finer structures such as photoreceptors. (**d**,**e**) are extracted and magnified areas of (**a**,**c**) to have a side-by-side comparison between configuration 1 and configuration 2 in (**f**) and (**g**). (**h**–**j**) Three-dimensional renderings of the areas in (**a**–**c**) B-scans are an average of five registered B-scans; volume data consist of 400 B-scans, each an average of four registered B-scans. The volume data were acquired using configuration 1.

With increasing number of averages the background noise is reduced and weaker signals of interest are enhanced: The dynamic range in the tomograms was measured by dividing the maximum value in the tomogram by the mean of the background noise. For the non-averaged tomogram a dynamic range of 17 dB was calculated, which increases in averaged tomograms to 24 dB, 29 dB and 30 dB for three, five and ten averages, respectively. In this setup, an averaging of five B-scans provided a good compromise between dynamic range and imaging speed as the choroid-sclera junction is well visible but the contrast does not improve significantly with ten averages or more. Further tomograms in this work will be presented as an average of five B-scans. The healthy subjects were imaged with all three sample arm configurations at various locations of the retina. A standard chin rest was used for more stable and reliable measurements.

Figure 4a–c,h–j show data acquired with a moderate FOV of approximately 15$^\circ$ using the telescope configuration 1. Figure 4a displays the retina in the region of the foveal pit where all individual layers are distinguishable. Sensitivity as well as axial resolution are even sufficient to visualize the external limiting membrane (ELM) with clear distinction from the other layers. The visibility of the optic nerve depression in Fig. 4b demonstrates sufficient sensitivity in depth, which allows contrasting the choroid-sclera junction. Figure 4c depicts the retina at a peripheral region, where more signal from the choroid layer is visible because the retina thickness is reduced in peripheral regions. Figure 4h–j show three-dimensional rendering in the respective locations foveal pit in Fig. 4d, optic nerve depression in Fig. 4e, and in a peripheral region in Fig. 4f.

**Figure 5:**
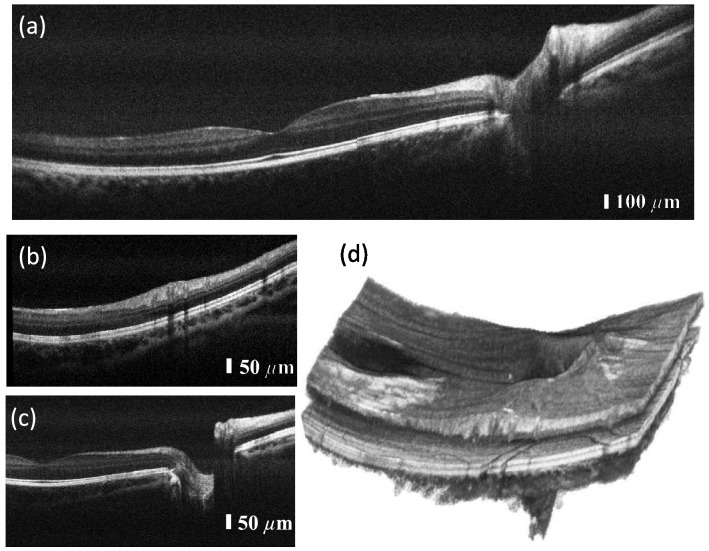
In vivo tomograms of a healthy retina with a larger FOV: (**a**) B-scan with a sampling of 800 A-scans per B-scan, average of five registered B-scans. (**b**,**c**) Selected B-scans of the acquired volume at different locations of the retina. Tomograms are an average of four registered B-scans. (**d**) Three-dimensional rendering of the acquired volume showing the foveal pit as well as the optic nerve depression in a single dataset.

In a SS-OCT system the acquisition time needed for a B-scan is mainly given by the speed of the swept source and the amount of sampling points (A-scans per B-scan). A multi-channel configuration as proposed in^16^, where multiple sample arms are acquired with multiple detection arms, would increase effective imaging speed, while keeping the same system sensitivity compared to a standard single channel configuration. Such a multi-channel configuration can be used in several ways, depending on the requirements: faster, wider FOV or finer lateral resolution/sampling. Considering a four-channel system, compared to a single-channel system the multiple sample arms could be used to acquire the same FOV as the single-channel system but four times faster since the FOV is scanned in parallel. The parallel sample arms could also scan a wider field in the same time though. Finally, the same amount of sampling/speed could be used to scan a moderate FOV but with higher lateral resolution. Configuration 1 is assumed to be the standard single channel configuration because it produces satisfactory imaging performance in terms of lateral resolution and FOV. Configuration 2 has comparably higher resolution, which requires a higher sampling density in order to benefit from the smaller focus point on the retina. The number of samples and, hence, the imaging speed was kept constant (800 A-scans per B-scan). The FOV was reduced by a factor of four because a four-channel configuration would scan the same area as configuration 1 in parallel. Figure 4f demonstrates imaging performance with configuration 2 in the foveal region and Fig.4g in a peripheral region of the retina. For a side-to-side comparison, the tomograms with the moderate FOV (configuration 1) in Fig. 4a,c were cropped and inserted in Fig. 4d,e. In both cases, the increased lateral resolution results in higher contrast for fine structures such as the external limiting membrane. In Fig. 4e, the photoreceptors start to show up, however not as clear as in Fig. 4g. A smaller depth of focus can be seen comparing Fig. 4g,e. To achieve a moderate FOV four parallel sample arms would be required to acquire the data with sampling parameters similar to the ones shown in Fig. 4a,c.

**Figure 6:**
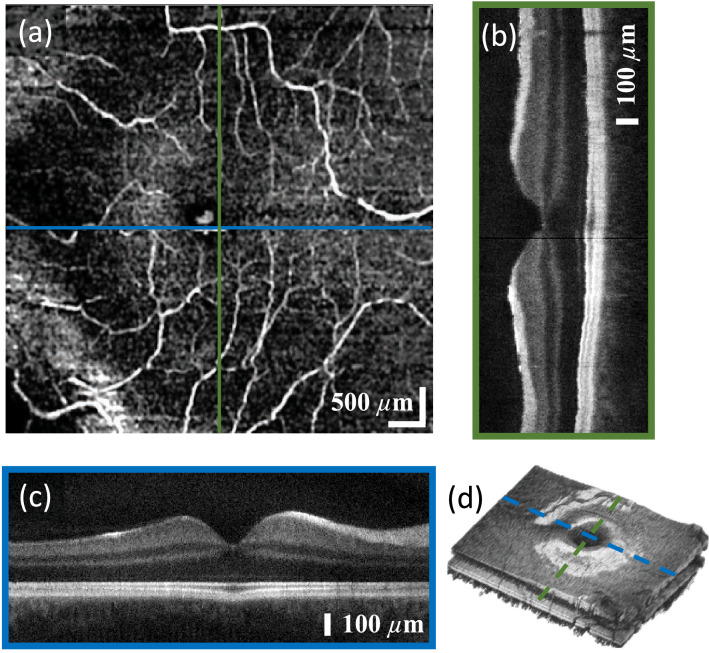
Angiographic data of a healthy volunteer. (**a**) Maximum intensity projection of the angiogram calculated from an acquired volume with a repetition of four consecutive B-scans. (**b**) Tomogram along the slow axis and (**c**) the fast axis from the (both four times averaged) (**d**) three dimensional intensity rendering of the volume.

A larger FOV is usually desirable in order to get an overall impression of the retina condition. In Fig. 5a, a large FOV covers the foveal pit as well as the optic nerve head in a tomogram acquired with 800 A-scan per B-scan, just as many as the previous B-scans in Fig. 4. The data in this figure were acquired with configuration 3, which has a beam diameter of 3.75 mm and a two-inch clear aperture enabling wide field imaging. For volume acquisition the scanning rates were reduced to 400 A-scans per B-scan and 400 B-scans per volume with a repetition number of four. Figure 5b,c show selected tomograms of the acquired volume at different slow-axis positions. While the more peripheral area in Fig. 5b contains several vessels of different diameters, the areas in or closer to the less dense foveal region in Fig. 5c show only few smaller vessels. However, in the region of the optic nerve depression larger vessels are well visible. Figure 5d shows a three-dimensional rendering of the acquisition, where the foveal pit and the optic nerve depression are fully visible.

Figure 6a demonstrates the visualization of the retinal vasculature. Volume data in the foveal region were acquired with four consecutive B-scans. Figure 6d presents the corresponding three dimensional rendering. From these data the complex OCTA was calculated. Figure 6c shows the central B-scan along the fast axis and Fig. 6b shows the central B-scan along the slow axis.

## Discussion

With an imaging speed of 50 kHz and eye safe laser powers (750 $\upmu$W) the presented PIC-based OCT system centered at 830 nm, based on an on-chip MZI achieves a system sensitivity of 94 dB, which is in the range of commercially used OCT systems. An axial resolution of 7.5 $\upmu$m in air was measured, which properly resolves all major individual layers of the retina. The measured spectra through the PIC further demonstrate the broadband capability of the waveguides and the on-chip building blocks, which are designed to support 100 nm bandwidth. The broadband capability of the PIC and its building blocks is discussed in detail in Nevlacsil et al.^16^. A low number of averaging further demonstrates a realistic clinical imaging scenario. The splitting ratio imbalance of the 50/50 coupler of 48:52 shows a deviation of approximately $5\%$ from the targeted design. In this specific setup it was possible to compensate the imbalance by aligning the dual balanced receiver towards an optimum balance. In general, tolerances of 3.5–6% are typical for fiber based 50/50 coupler that are used for OCT applications, hence the splitting ratio deviation of the on-chip 50/50 coupler lies within typical tolerances of OCT components.

Three sample arm configurations, i. e. combinations of scanning and ophthalmic lenses, were used to compare the imaging performances of the system. Configuration 1, with a beam diameter of 2 mm at the cornea, gives moderate FOV as well as satisfying resolution. A 4 mm laser beam diameter at the cornea (configuration 2) produces tomograms with finer lateral resolution and, with the same amount of sampling, finer details such as photoreceptors are resolvable. In a potential multi-channel system four parallel sample arms could be used to produce tomograms with a moderate FOV (e.g. configuration 1, Fig. 4a–c) and a lateral resolution comparable to the results in Fig. 4i,j (configuration 2) without sacrificing sensitivity or time needed for the acquisition. On the other hand, multiple parallelized sample arm beams could produce wide FOV tomograms with a resolution as well as imaging speed of configuration 1. Figure 5a shows the retina with a larger FOV (3.75 mm), including the foveal pit as well as the optic nerve head, which was acquired in one setting using configuration 3. With a multi-channel configuration a higher dynamic range and a finer resolution could be achieved with larger FOVs. In terms of laser safety, the standards ISO 15004 and ANSI Z80.36 allow 500 $\upmu$W per beam on the cornea with up to 16 parallel beams. While the individual parallel beams are focused on different spots on the retina, the beams may overlap in the cornea^16^. The acquisition of three-dimensional data, which is required to calculate the OCTA, was demonstrated with a moderate FOV in Fig. 4h–j as well as with a larger FOV in Fig. 5d. A good sensitivity in the non-averaged data is required for B-scans to be registered to one another computationally, which was achieved with this PIC-based OCT system as shown in the non-averaged tomogram in Fig. 3b. From the three-dimensional renderings it can be seen that registration worked well, also for a larger FOV. From the three-dimensional data in Fig. 5d several examples of B-scans with 400 A-scans per B-scan were shown in Fig. 5b–d. Although the sampling is reduced, individual layers can still be distinguished.

OCTA in a healthy subject was demonstrated as well. The angiogram in Fig. 6a shows vessels in the foveal region and no signal in the macula as expected^18^.

Insertion losses of approximately 3.4 dB with one fiber-PIC coupling event and 6.7 dB when two fiber-PIC coupling events are involved, respectively, are the main factor for reduced system sensitivity, compared to a fiber-based OCT system. The usage of lensed fibers could increase coupling efficiency by a factor of two. The usage of $\lambda$/4 plates, which typically have a better broadband performance compared to polarization paddles could improve coupling of broader bandwidth light. Although the presented setup uses a miniaturized MZI on the PIC, in terms of overall size it is still comparable with state-of-the-art systems and a true miniaturization has not been achieved yet. However, the presented PIC-based OCT system is, to the best of our knowledge, the first one capable of in vivo retinal imaging using an on-chip MZI with an imaging speed, system sensitivity and axial resolution that might be useful for clinical application. These results are an important step towards a full PIC-based SS-OCT system, which could facilitate the development of miniaturized OCT: the compatibility with already well established CMOS processes enables the fabrication of an opto-electronic OCT device with high integration density in one fabrication plant. On the one hand, a high integration density can be achieved in the photonic part, as multiple building blocks, such as couplers with various splitting ratios or PBS can be realized in one fabrication step and the need for post-fabrication packaging is significantly reduced. On the other hand, a high level of integration of the full electronic acquisition and processing chain on the same PIC can be realized. Once fabrication processes are established, the realization of multiple sample and detection channels will be easier and more cost effective, compared to state-of-the-art fiber-based systems, while a significantly smaller footprint can be maintained. The costs of individual opto-electronic PICs is reduced since several dozens of PICs can be fabricated in parallel and multiple functionalities could be realized at one fabrication site. While CMOS-based detection and pre-processing electronics are well-established, the design, fabrication and testing of CMOS-compatible photonics, suitable for in vivo retinal OCT imaging, is an important step towards a combined opto-electronic PIC-based OCT system. The integration of on-PIC light sources^19^ will further increase system compactness and programmable PICs^20^ might increase flexibility.

## Methods

### Photonic integrated circuit

The core element of the OCT system is the on-chip MZI. Silicon nitride (SiN) optical waveguides with silicon dioxide (${\text {SiO}}_2$) as cladding material support the wavelength region around 840 nm, which makes them well suited for ophthalmic OCT imaging. Figure 1d depicts the layout of the on-chip MZI. It consists of a power splitting coupler to supply laser light to the sample and reference arm in TE mode. A polarization beam splitter (PBS) in each arm forwards the backreflected light in TM mode to the on-chip 50/50 coupler, which acts as a 50/50 coupler, where light from both arms is interfered. To achieve a rotation of the light polarization by 90$^\circ$ the light can be passed through a 45$^\circ$ rotated $\lambda$/4 plate outside of the PIC. In the first pass the linear polarization of the light is changed to circular polarization and after the reflection the circular polarized light passes the $\lambda$/4 plate a second time resulting in a 90$^\circ$ rotated polarization compared to the emitted polarization from the PIC. A polarization controller can be used to substitute the functionality of the 45$^\circ$ rotated $\lambda$/4 plate to allow for an easier implementation with a more limited wavelength bandwidth. However, the measured axial resolution indicates that the full bandwidth was supported using polarization paddles.The interfered light is then forwarded to the PIC end facet, where photodiodes pick up the signal for dual balanced detection. Waveguides with a cross section of $(700 \times 160)\;{\text {nm}}^2$ exhibit propagation losses of 1 dB/cm and 0.5 dB/cm for TE-like and TM-like polarization, respectively. A detailed description of the design, fabrication steps and characterization measurements is given in^16^. Figure 1a shows a photograph of the fabricated PIC.

### PIC-based OCT setup

Figure 1d shows a schematic of the OCT measurement setup. Light from a swept source with 56 nm bandwidth centered at 830 nm (prototype, EXALOS AG, Switzerland) is amplified by a broadband booster amplifier (prototype, EXALOS AG, Switzerland) resulting in powers of up to 50 mW ex fiber. Three 3-axis piezo flexure stages (MDT630B, Thorlabs Inc., USA) are used to align the single mode fibers (P3-780Y-FC-2) to the input, sample and reference ports of the PIC. The input port guides the light towards the on-chip power splitter, which then forwards according portions of light to the two arms: The power splitting ratio between sample path and reference path is 95:5. At the sample arm port the light is coupled to a single mode fiber. The fiber is connected to a reflective fiber collimator (RC02 APC-P01, Thorlabs Inc., USA), which forwards the light in free-space to galvanometric scanners (621 OH Cambridge Technology, USA). Three different telescope configurations consisting of two lenses for the projection of the beam on the cornea were tested (see Table 2): Configuration 1 projects the 2 mm beam with a magnification of 1 (two 75 mm lenses, AC508-075-B, Thorlabs Inc. USA) onto the cornea. In configuration 2, the beam diameter is magnified to 4 mm at the cornea (Lens 1: AC508-150-B, Thorlabs, Lens 2: AC508-075-B, Thorlabs) for higher lateral resolution. Configuration 3 uses a custom-made prototype ophthalmic lens, which was provided by ZEISS with a focal length of 45 mm and a lens diameter of two inches, which results in 3.75 mm beam diameter at the cornea, and a clear aperture of approximately two inches, which represents an optical design that enables wide field scanning.

**Table 2:** Overview of the three sample arm configurations: different focal lengths (*f*) of the two lenses in the telescope results in different beam diameters (*Dia.*) on the cornea and field of views (*FOV*).

Configuration	f lens 1 (mm)	f lens 2 (mm)	Dia. on cornea (mm)	FOV ($^\circ$)	lat. res. ($\upmu$m)
1	75	75	2	15	11.9
2	150	75	4	4	5.95
3	75	45	3.75	45	6.35

The lateral resolution (*lat. res.*) was calculated assuming a focal length of the eye with 22 mm.

The reference arm consists of a fiber collimator (F220 APC-850, Thorlabs Inc., USA), a focusing lens (AC245-50-B, Thorlabs Inc., USA) as well as a silver coated mirror. Manual polarization controllers (FPC030, Thorlabs Inc., USA) are used in each arm to match the polarization to one another and to turn the polarization for the polarizing beam splitter to forward the light towards the 50/50 coupler. Backreflected light from both arms is coupled back onto the chip with a 90$^\circ$ rotated polarization, where the polarization beam splitters forward the light in TM polarization to an on-chip 50/50 coupler. The TE/TM polarization of the light on the PIC was calibrated by optimizing the polarisation paddle positions to maximum output power. The interfered light exits the PIC at the end-facet via two ports and the two beams are collected by a dual balanced receiver (prototype, 45 MHz bandwidth, EXALOS AG, Switzerland) with the photodiodes being mounted to match the distance of the output waveguides. Figure 1b shows a photograph of the centerpiece of the setup: The PIC with dimensions of $(12 \times 5)\;{\text {mm}}^2$ is mounted on a post and is surrounded by comparably large standard setup components. Figure 1c shows the PIC with the on-chip MZI and the fibers as well as the dual balanced receiver.

### Data acquisition and post processing

Data was acquired using LabView (Version 18.0, 64 bit, National Instruments, USA). Data from the dual balanced detector are collected on the computer via a data acquisition device (ATS9350, AlazarTech, Canada). The second port of the data acquisition card collects the reference interference signal provided by the swept source. The A-scan trigger of the latter clocks the acquisitions. Synchronization between scanning and acquisition is achieved using an FPGA (NI PCIe 7842R, National Instruments, USA). Image reconstruction was done using Matlab (R2019a, 9.6.1135713, Mathworks, USA): The acquired data is mapped to k-space and dispersion is removed using the method in^21^. Background removal is achieved by subtracting the mean of a tomogram from every A-scan. Fourier transformation of the spectrum results in the depth resolved A-scans.

At a depth of approximately 700 $\upmu$m an electrical noise signal occurs with an origin that could not be determined fully but most likely comes from insufficient electrical shielding of the customized dual balanced receiver. In order to collect the light directly from the PIC end facet, the photodiodes of a commercial receiver were mounted on an additional plate (see yellow part in Fig. 1c). For this reason, the housing of the receiver has an opening, which could be the source of electrical noise in the tomograms. Since the noise is also present when the receiver is not assembled in the OCT system the PIC can be excluded as source of origin. As the electrical noise is not constant over time, standard background subtraction does not remove this line. For data in which the retinal signal is at the same depth location as the noisy line we implemented a selective background removal procedure, which calculates the correlation of the noise in different B-scans and subtracts the mean background of B-scans with similar noise patterns. This technique works well for data sets consisting of more B-scans. For data consisting of less B-scans a manual line removal was achieved by line wise adaption of intensity values.

The reconstructed B-scans were finally loaded into ImageJ, in which B-scan registration using the translation method registration function of the Strackreg Plugin and image averaging were performed. Three-dimensional visualization of data sets was created using Fiji’s Volume Viewer Plugin.

For the OCTA calculation, four corresponding B-scans per slow axis position were acquired. An adapted version of the processing pipeline documented in Ref.^22^ was used to extract angiographic data. The complex valued B-scans within a set of four B-scans were aligned with respect to the first B-scan in the set in both, the fast axis and depth directions. The average phase difference between consecutive B-scans was calculated in order to compensate for bulk motion. The data was then thresholded by an empirically determined intensity value. Finally, pairwise differences among the four bulk-motion-corrected complex B-scans at each slow axis position were computed. The average of the absolute values from these was computed as one angiographic B-scan for every set of recorded slow axis positions.
